# Insulin delivery characteristics and glycemic control in older adults with type 1 diabetes using the Minimed™ 780G system: a real-world analysis

**DOI:** 10.3389/fendo.2026.1910192

**Published:** 2026-08-07

**Authors:** Špela Volčanšek, Petra Vidmar, Andrej Janež, Aleš Skvarča

**Affiliations:** 1 University Medical Centre Ljubljana, Department of Endocrinology, Diabetes and Metabolic Diseases, Ljubljana, Slovenia; 2 Faculty of Medicine, University of Ljubljana, Ljubljana, Slovenia

**Keywords:** advanced technologies, automated insulin delivery, hypoglycemia, individualized care, older adults, real-world data, time in tight range, type 1 diabetes

## Abstract

**Introduction:**

Diabetes self-management in older adults with type 1 diabetes (T1D) presents a unique clinical challenge, since maintaining tight glycemic control must be carefully weighed against age-related vulnerabilities. This study aimed to analyze glycemic control through the effects of customized algorithm settings in a cohort of older adults with T1D using an automated insulin delivery (AID) system.

**Methods:**

This retrospective real-world data analysis included users of the MiniMed™ 780G AID system, aged ≥60 years. Continuous glucose monitoring (CGM) data including time in range (TIR; 70–180 mg/dL; 3.9–10.0 mmol/L), time in tight range (TITR; 70–140 mg/dL; 3.9–7.8 mmol/L), time below range (TBR; <70 mg/dL; <3.9 mmol/L), level 2 TBR (<54 mg/dL; <3.0 mmol/L) and time above range (TAR; >180 mg/dL; >10.0 mmol/L) were analyzed alongside algorithm settings and daily insulin use. Group comparisons utilized standard parametric and non-parametric statistical tests. A multivariable logistic regression model identified independent clinical predictors of high glycemic performance (TITR ≥50%).

**Results:**

The cohort comprised 97 AID users (70.1% female, mean age 67.9 ± 6.7; range 60–90 years). Overall glycemic control was good, with mean TIR 76.1%, TITR 51.1% and TBR 1.1%. Fourteen participants (14.4%) used the manufacturer’s recommended optimal settings (ROS); i.e., target glucose of 5.5 mmol/L and active insulin time of 2 hours. CGM metrics did not differ between ROS and non-ROS users for TIR, TITR and TBR. When stratifying the cohort by glycemic performance, 55 participants (56.7%) reached TITR ≥50%; however, the adoption of ROS in high (≥50%) TITR and lower (<50%) TITR subgroups was similar (16.4% vs 11.9%; Fisher p=0.58). When compared to the lower TITR subgroup, participants in the high TITR subgroup delivered less auto-correction insulin (14.2% vs 20.6%, p<0.001) and a higher proportion of manual bolus insulin (46.1% vs 35.2%, p<0.001), which remained a significant predictor of high TITR after multivariable adjustment. TBR was significantly higher in the high (≥50%) TITR subgroup, although it remained low in absolute terms (1.5% vs. 0.8%; p=0.017).

**Conclusion:**

While the MiniMed™ 780G AID system use is associated with low CGM-derived hypoglycemia exposure and highly effective glycemic control, regardless of customized settings, superior glycemic control is associated with a higher proportion of user-initiated manual boluses. The algorithm supports, but cannot replace, user engagement.

## Graphical Abstract

**Figure:**
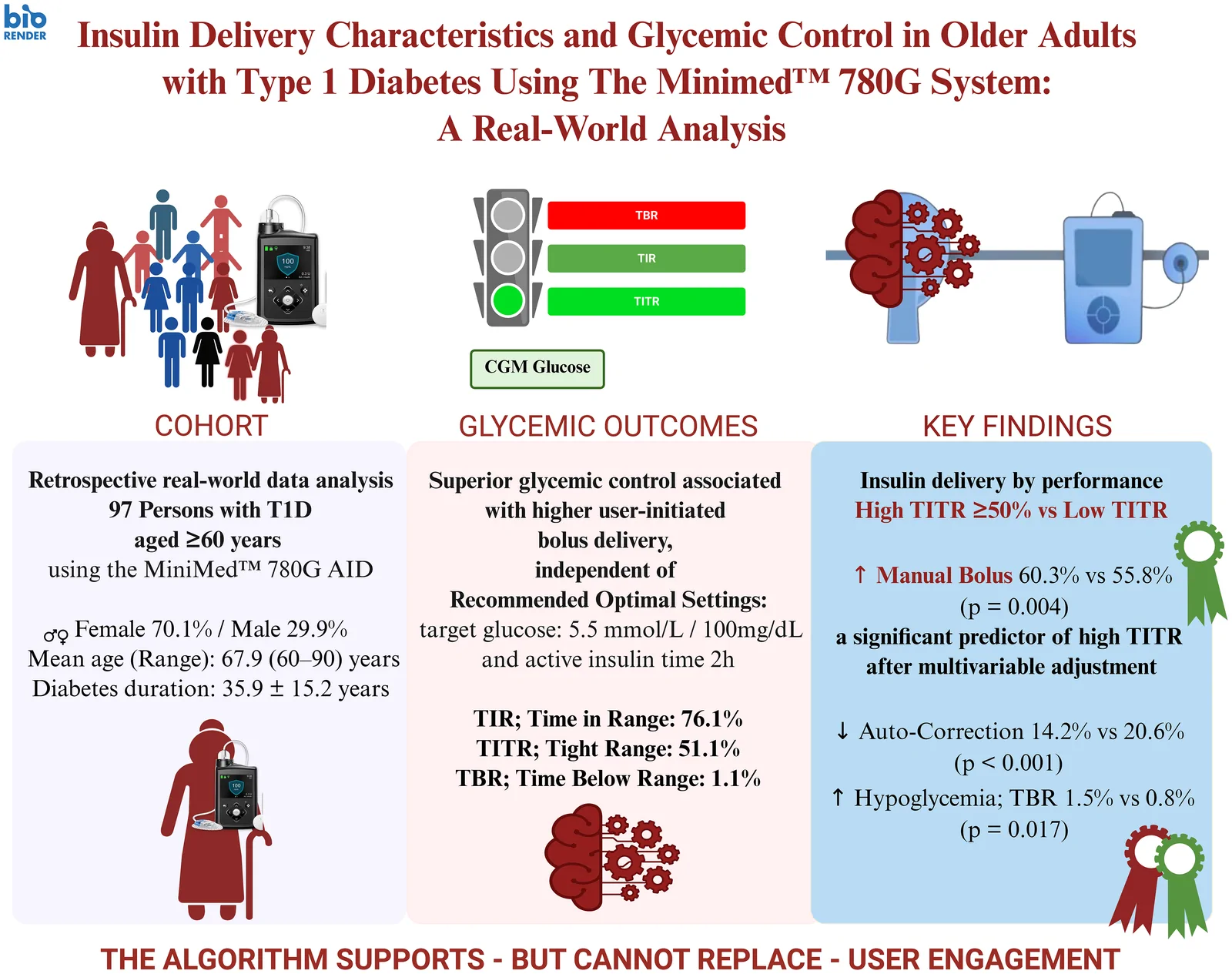

## 1 Introduction

The demographics of type 1 diabetes (T1D) are changing, with many older adults now living with the disease and using diabetes technology (1–3). About 20% of people with T1D are aged 60 or above, and this is expected to rise in resource-limited as well as in high-income countries (4, 5). This change results from a longer life expectancy among those diagnosed in childhood and improved recognition of adult-onset autoimmune diabetes (5). Data show over 50% of T1D diagnoses happen after age 20, challenging the view of T1D as only a childhood disease (1, 3). Older adults with T1D often have age-related comorbidities, cognitive issues, visual impairments, and reduced manual dexterity, complicating self-management and technology use (6–8). The disease burden shifts from family support in childhood to independence in adulthood and sometimes back to assisted care in old age (1, 7, 9, 10). These factors highlight that T1D management should be personalized based on age, comorbidities, and, most importantly, functional status (7, 9, 11).

Despite these challenges, the adoption of diabetes technology among older adults has increased substantially (8, 12). Randomized controlled trials have demonstrated the advantages of automated insulin delivery (AID) systems in hypoglycemia risk reduction and improved glycaemic control in older adults with T1D compared with sensor-augmented insulin pump therapy or manual insulin delivery (13–16). However, physical limitations, including impaired vision, reduced manual dexterity, and cognitive decline, may hinder the use of AID, reinforcing clinical caution when applying more aggressive treatment strategies in older adults (1, 11). A particularly important concern in older adults with T1D is their increased vulnerability to severe hypoglycemia and hypoglycemia unawareness (9, 17–19). Consequently, current guidelines recommend individualized glycaemic targets in older adults, often prioritizing safety and avoidance of hypoglycemia over tight glycemic control (1, 20). Fear of hypoglycemia frequently leads to de-intensification of therapy, including higher glucose targets, which may result in suboptimal glycemic control (11, 12, 17).

Large real-world analyses have demonstrated that use of the MiniMed™ 780G system, which automatically adjusts basal insulin delivery and administers correction boluses (21–26), is associated with improved glycemic control and reduced exposure to hypoglycemia in broad T1D populations (21–25, 27, 28). The manufacturer’s generally “recommended optimal settings” (ROS) for this system include an active insulin time (AIT) of 2 hours and a target glucose (TG) of 100 mg/dL (5.5 mmol/L) (21–25); settings considered relatively aggressive. Consequently, safety concerns continue to drive substantial clinical hesitation regarding the adoption of these settings in older adults.

Therefore, this study aimed to investigate whether the use of ROS in MiniMed™ 780G users aged 60 or older is associated with effective glycaemic control, as assessed by time in tight range (TITR), without increasing the hypoglycemia exposure. Additionally, we explored the role of insulin-delivery characteristics.

## 2 Methods

### 2.1 Study design and data source

Eligible participants were current users of the MiniMed™ 780G AID system. They had shared their device data via cloud-based connectivity during or shortly before the study period, enabling retrieval through the CareLink™ clinic platform. Participants’ CareLink™ personal data during the period from October 1st, 2025, until March 31st, 2026, were used to examine continuous glucose monitoring-based endpoints and insulin delivery in users aged 60 years and above attending the outpatient clinic of the Department of Endocrinology, Diabetes and Metabolic Diseases, University Medical Centre Ljubljana, Slovenia. Retrospective data were extracted from the CareLink database over a 30-day window, defined as the 30 consecutive days immediately preceding each participant’s most recent clinical visit or the most recent data upload while visiting nurse educators. Participants were excluded if they failed to meet >70% active CGM wear time threshold or if their CareLink reports lacked sufficient data for the primary glycaemic and behavioral metrics (29). GlyCulator 3.0 (30) was used to extract the raw CareLink data. Application of these criteria resulted in the exclusion of 17 AID users, yielding a final analytical cohort of N = 97.

The data were retrieved in accordance with data protection regulations, and all information on persons with diabetes (PwD) was collected in a fully anonymized format from the database, ensuring that individual identities were protected throughout the analysis, as approved by the Slovenian National Medical Ethics Committee (No. 0120-464/2025-2711-3).

Glycaemic outcomes were derived from continuous glucose monitoring data and included time in range (TIR; 70–180 mg/dL; 3.9-10.0 mmol/L), time in tight range (TITR; 70–140 mg/dL; 3.9-7.8 mmol/L), time below range (TBR; <70 mg/dL; <3.9 mmol/L), level 2 TBR (<54 mg/dL; <3.0 mmol/L) and time above range (TAR; >180 mg/dL; >10.0 mmol/L). Level 2 TAR (>250 mg/dL; >13.9 mmol/L) could not be reported, because the data-extraction protocol was calibrated to capture TITR and the software binning parameters precluded its simultaneous extraction.

Baseline clinical and therapeutic characteristics were recorded, including diabetes duration, body mass index (BMI), body weight, total daily insulin dose (TDD), TDD per body weight, the duration of insulin-pump (continuous subcutaneous insulin infusion; CSII) and AID therapy. Diabetes duration, CSII duration, and AID therapy duration were calculated relative to 2026 from the year of diagnosis, CSII initiation and AID initiation, respectively. Where a variable was not recorded for a given participant, that participant was excluded from the corresponding summary only. Based on retrospective CareLink data classifications, ‘manual boluses’ were defined as all user-commanded bolus events, i.e., carbohydrate-matched meal boluses, explicitly excluding the automated, algorithm-driven correction boluses characteristic of the 780G system.

Participants were stratified according to two predefined comparisons. First, glycaemic outcomes were compared between users of ROS, defined as an AIT of 2 hours and a TG of 100 mg/dL (5.5 mmol/L), and participants not using this combination of settings. Second, to assess system efficacy and evaluate potentially increased hypoglycaemia exposure, participants were further categorized based on TITR (70–140 mg/dL; 3.9–7.8 mmol/L) into a high-performance subgroup with TITR ≥50% and a lower-performance subgroup with TITR <50% (31, 32).

### 2.2 Statistical analysis

Continuous variables are presented as mean ± SD or mean with 95% confidence intervals (95% CI), and categorical variables as counts and percentages. The distribution of continuous variables was assessed for normality using the Shapiro–Wilk test. Between-group comparisons of continuous variables were performed using Welch’s unequal-variance t-test or the Mann–Whitney U test, as appropriate, while categorical variables were compared using Fisher’s exact test. To identify independent predictors of high glycemic performance (TITR ≥50%), a multivariable logistic regression model was fitted, including manual bolus proportion, age, sex, total daily insulin dose, diabetes duration and AID duration; adjusted odds ratios (OR) with 95% CI are reported. Because manual bolus, auto-correction and basal proportions are mutually dependent (a higher manual bolus proportion is accompanied by fewer algorithm-driven auto-corrections), only manual bolus was entered to avoid collinearity. Coefficient of variation (%CV) and GMI were not entered as predictors, as they reflect glycemic outcomes rather than independent clinical or behavioral factors. Subgroup analyses were conducted by stratifying the cohort according to relevant demographic and clinical characteristics. All statistical tests were two-sided, and a P value <0.05 was considered statistically significant. Statistical analyses and figure generation were performed in Python using the Polars, SciPy, statsmodels, and Matplotlib libraries.

## 3 Results

### 3.1 Study cohort

Our study cohort comprised 97 AID users living with T1D (70.1% female, 29.9% male; age range 60–90 years; mean age 67.9 ± 6.7). Their overall glycemic control was good, with a mean TIR of 76.1%, TITR 51.1%, TBR 1.1%, and TAR 22.6%. Level 2 TBR (<3.0 mmol/L) was highly limited across the cohort at 0.2%. The mean %CV was 31.3%, and the mean GMI was 6.9%. Baseline characteristics, including stratification by TITR subgroup, are summarized in **Table 1**. Males exhibited a slightly higher mean TIR (78.3%) compared to females (75.7%), but this difference was not statistically significant (p=0.163). The system operated with equal efficacy across both sexes.

**Table 1:** Baseline characteristics of the study cohort, stratified by TITR subgroup.

Characteristic	Overall (n = 97)	TITR ≥ 50% (n = 55)	TITR < 50% (n = 42)	P
Age, years	67.9 ± 6.7	66.9 ± 6.6	69.5 ± 6.7	0.062
Sex, female, n (%)	68 (70.1)	39 (70.9)	29 (69.0)	1.000†
BMI, kg/m^2^	26.4 ± 4.6	26.0 ± 4.8	26.9 ± 4.5	0.380
Diabetes duration, years	35.9 ± 15.2	34.9 ± 15.6	37.3 ± 14.7	0.478
CSII duration, years	12.1 ± 5.9	11.8 ± 5.5	12.5 ± 6.4	0.598
AID duration, years	2.9 ± 1.2	2.7 ± 1.3	3.1 ± 1.1	0.058
TDD, U/day	40.2 ± 19.1	39.2 ± 17.0	41.4 ± 21.7	0.588
TDD per body weight, U/kg/day	0.53 ± 0.19	0.53 ± 0.18	0.54 ± 0.20	0.874
GMI, %	6.89 ± 0.30	6.70 ± 0.16	7.14 ± 0.26	<0.001
%CV	31.3 ± 4.4	30.7 ± 4.3	32.2 ± 4.5	0.103

Data are mean ± SD unless indicated. P values are from Welch’s t-test for continuous variables and †Fisher’s exact test for sex. Diabetes duration, CSII duration, and AID duration were calculated to 2026 from the year of diagnosis, CSII initiation, and AID initiation, respectively. Summaries are over available data: diabetes duration n=85, TDD n=96, TDD per body weight n=86, and BMI, CSII duration, and AID duration n=95. TITR, time in tight range (70–140 mg/dL; 3.9–7.8 mmol/L); CSII, continuous subcutaneous insulin infusion; AID, automated insulin delivery; BMI, body mass index; %CV, coefficient of variation; GMI, glucose management indicator; SD, standard deviation; TDD, total daily insulin dose.

On average, PwD received 58% of their total daily insulin as a bolus and 42% as basal insulin. The mean proportion of bolus insulin delivered via auto-correction was 17%. This parameter exhibited the highest inter-individual variability — in some PwD, the system delivered only 5% of bolus insulin via auto-corrections, whereas in extreme cases, it accounted for nearly 32%.

### 3.2 ROS users and non-users

Of all participants in our study cohort, fourteen participants (14.4%) were using ROS. We found no statistically significant difference in CGM metrics between users and non-users of ROS for TBR, Level 2 TBR, TITR, TIR or TAR, nor in GMI. hypoglyceamia exposure, assessed by TBR and Level 2 TBR, was low in both subgroups (**Table 2**).

**Table 2:** CGM metrics in Recommended Optimal Settings (ROS) users versus non-users.

Metric	ROS (n = 14)	Non-ROS (n = 83)	P-value
TAR [%]	24.9 (19.0–30.7)	22.3 (20.3–24.3)	0.453
TIR [%]	74.1 (68.4–79.8)	76.4 (74.5–78.3)	0.472
TITR [%]	49.9 (44.8–54.9)	51.3 (49.1–53.6)	0.608
TBR [%]	1.1 (0.2–1.9)	1.2 (0.9–1.5)	0.561
Level 2 TBR [%]	0.1 (0.0–0.4)	0.2 (0.1–0.2)	0.952
GMI [%]	7.0 (6.7–7.2)	6.9 (6.8–6.9)	0.528

Values are mean (95% CI). Recommended Optimal Settings (ROS): target glucose (TG) 5.5 mmol/L (100 mg/dL) and active insulin time (AIT) 2 h. P values from Welch’s t-test. TAR, time above range (>180 mg/dL; >10.0 mmol/L); TIR, time in range (70–180 mg/dL; 3.9–10.0 mmol/L); TITR, time in tight range (70–140 mg/dL; 3.9–7.8 mmol/L); TBR, time below range (<70 mg/dL; <3.9 mmol/L); Level 2 TBR (<54 mg/dL; <3.0 mmol/L) and GMI (Glucose management indicator).

### 3.3 TITR-based subgroup analysis

To gain a more detailed insight into metabolic control, the cohort was stratified into two subgroups based on the achievement of TITR: a high (≥50%) TITR subgroup (n=55) and a lower (<50%) TITR subgroup (n=42). The baseline characteristics table (**Table 1**) stratifies the cohort by the two TITR subgroups and provides statistical comparisons between them.

#### 3.3.1 Settings comparison between the high (≥50%) TITR subgroup and the lower (<50%) TITR subgroup

The prevalence of ROS use did not differ significantly between the two subgroups, with ROS adopted by 16.4% of participants in the high (≥50%) TITR subgroup and 11.9% of PwD in the lower (<50%) TITR subgroup (p=0.58).

Within the high (≥50%) TITR subgroup, insulin delivery settings clustered around an AIT of 2.5 hours rather than the generally recommended 2 hours; however, TG of 5.5 mmol/L was most frequently used. In contrast, the lower (<50%) TITR subgroup exhibited greater variability in both AIT and TG settings, including longer AIT values and a higher TG.

#### 3.3.2 Glycemic control by TITR subgroup

Across both TITR subgroups, clear differences in glycemic control were observed. The high (≥50%) TITR subgroup also achieved significantly higher overall TIR (mean 82.6%) compared to the lower (<50%) TITR subgroup with mean TIR of 69.9% (p<0.001). In addition, the lower (<50%) TITR subgroup spent almost twice as much time above range as compared with the high (≥50%) TITR subgroup (mean 29.3% vs. 15.9%; p<0.001). In contrast, TBR was significantly higher in the high (≥50%) TITR subgroup, although it remained low in absolute terms (1.5% vs. 0.8%; p=0.017).

#### 3.3.3 Insulin distribution and algorithm response by TITR subgroup

**Figure 1:**
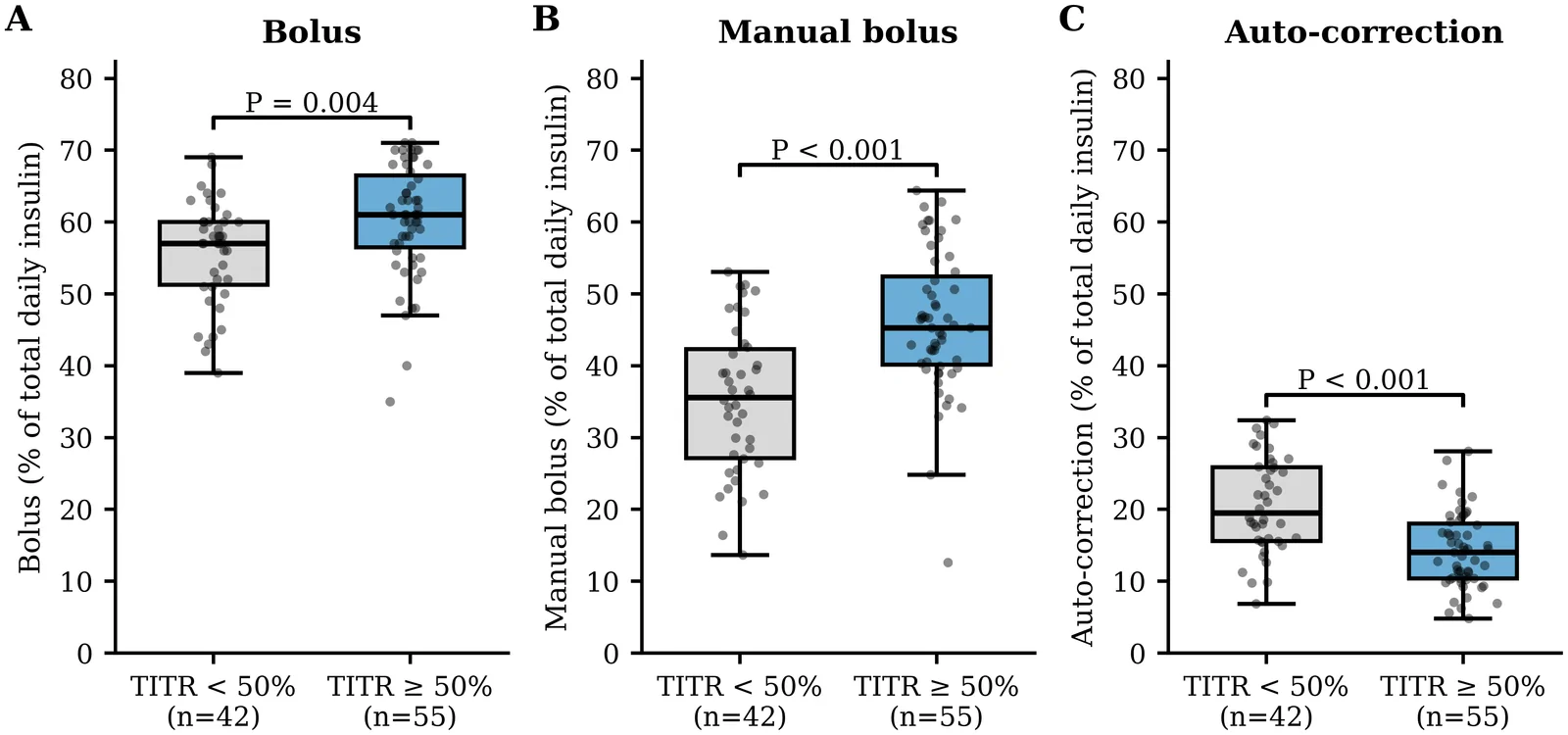
Comparison of insulin delivery composition between the high (≥50%) TITR subgroup and lower (<50%) TITR subgroup: Box plots showing the distribution of **(A)** bolus insulin as a proportion of total daily insulin, **(B)** manual bolus insulin as a proportion of total daily insulin, and **(C)** auto-correction insulin as a proportion of total daily insulin. Horizontal lines indicate medians, boxes represent the interquartile range, whiskers indicate the range within 1.5 × IQR, and points represent individual observations, and p-values are calculated via Welch’s t-test. Manual Bolus: A user-initiated dose of rapid-acting insulin delivered to cover carbohydrate intake or to manually correct hyperglycaemia. Auto-correction: Algorithm-driven, automated insulin delivery designed by the closed-loop system to correct rising or elevated glucose levels without requiring user intervention. TITR, time in tight range (70–140 mg/dL; 3.9–7.8 mmol/L.

Regarding insulin distribution and algorithm response, the high (≥50%) TITR subgroup required significantly more bolus insulin as well as significantly less auto-correction insulin when compared to the lower (<50%) TITR subgroup (**Table 3**). Specifically, in the high (≥50%) TITR subgroup versus lower (<50%) TITR subgroup, bolus insulin accounted for 60.3 (58.2–62.4) versus 55.8 (53.6–58.0) of total daily insulin (P = 0.004), comprising manual bolus (46.1 (43.4–48.7) versus 35.2 (32.0–38.4); P <0.001) and auto-correction (14.2 (12.8–15.6) versus 20.6 (18.5–22.7); P <0.001), accordingly. These differences were further reflected in the distribution of insulin delivery components, as visualized using box plots (**Figure 1**). In a multivariable logistic regression for high TITR (≥50%), a higher manual bolus proportion (adjusted OR 1.20 (1.10–1.30) per 1%; P < 0.001) was independently associated with high TITR, whereas age, sex, total daily insulin dose, diabetes duration and AID duration were not (**Table 4****).**

**Table 3:** Comparison of bolus insulin delivery between the high (≥50%) and lower (<50%) TITR subgroups.

Metric	TITR ≥ 50% (n=55)	TITR < 50% (n=42)	P-value
Bolus insulin, % of total daily insulin	60.3 (58.2–62.4)	55.8 (53.6–58.0)	0.004
Manual bolus, % of total daily insulin	46.1 (43.4–48.7)	35.2 (32.0–38.4)	<0.001
Auto-correction, % of total daily insulin	14.2 (12.8–15.6)	20.6 (18.5–22.7)	<0.001

Values are mean (95% CI) and P values from Welch’s t-test. Basal insulin (the structural complement of bolus) is not shown. TITR, time in tight range (70–140 mg/dL; 3.9– 7.8 mmol/L).

**Table 4:** Multivariable logistic regression: independent predictors of high glycemic performance (TITR ≥ 50%).

Predictor	β (SE)	Adjusted OR (95% CI)	P
Manual bolus, % of total daily insulin	0.180 (0.043)	1.20 (1.10–1.30)	<0.001
Age, years	-0.044 (0.040)	0.96 (0.88–1.04)	0.275
Female sex (vs male)	0.856 (0.720)	2.35 (0.57–9.66)	0.235
Total daily insulin dose, U/day	0.016 (0.018)	1.02 (0.98–1.05)	0.360
Diabetes duration, years	-0.006 (0.019)	0.99 (0.96–1.03)	0.761
Automated insulin delivery (AID) use duration, years	-0.076 (0.242)	0.93 (0.58–1.49)	0.753

Logistic regression for high TITR (≥50%), fitted on complete cases (n=84; 48 events), pseudo-R2=0.32. Only manual bolus is entered among insulin-delivery metrics (manual bolus and auto-correction are inversely related). CV and GMI were not entered as they reflect glycemic outcomes rather than independent predictors. TITR, time in tight range (70–140 mg/dL; 3.9–7.8 mmol/L); CV, coefficient of variation; GMI, glucose management indicator.

## 4 Discussion

In this real-world cohort of older adults with T1D aged 60 years or above using the MiniMed™ 780G AID system, the adoption of ROS was not associated with increased risk of hypoglycemia, as TBR remained low and comparable between ROS users and non-users. On the other hand, the use of ROS failed to further improve glycemic control in our study cohort. Moreover, our data suggest that achieving superior metabolic outcomes - characterized by a high TITR – is associated with greater PwD engagement. Specifically, achieving a higher TITR was associated with a greater proportion of user-initiated bolus insulin compared with system-driven auto-corrections. This finding suggests the ongoing necessity of next-generation algorithms, capable of autonomously detecting and aggressively managing mealtime excursions without user input.

Our findings support low CGM-derived hypoglycemia exposure with more aggressive algorithm settings in selected older adults, potentially reducing the need for overly conservative settings driven by fear of hypoglycemia. Randomized controlled data demonstrate that AID reduces hypoglycemia exposure in older adults compared with sensor-augmented pump therapy or multiple daily insulin injections (14, 33). Similarly, hybrid closed-loop therapy has been shown to reduce TBR without compromising glycaemic control in adults with T1D, even when compared with optimized manual therapy (33). Current clinical guidelines emphasize individualized glycaemic targets in older adults, prioritizing hypoglycaemia avoidance because of its association with falls, cognitive decline, and cardiovascular morbidity (1, 11). This interpretation aligns with emerging real-world evidence demonstrating variability in optimal AIT settings among MiniMed™ 780G users, with longer AIT associated with improved stability in selected populations (21–25, 27). This was also observed in our study population. Current diabetes technology guidelines and strategies to optimize care emphasize the need for individualized AID parameter optimization rather than uniform application of manufacturer defaults, particularly in populations with altered physiology, such as older adults (1, 34). Importantly, the MiniMed™ 780G algorithm responds to glucose trends rather than chronological age (19–23). Older adults face the highest risk of severe hypoglycaemia (12), and our findings support the concept that AID may effectively “flatten” age-related disparities in glycaemic outcomes, bringing this traditionally challenging population closer to the glycaemic stability observed in middle-aged adults (27).

Despite the favorable safety profile of ROS, their use was not associated with superior glycaemic control as measured by TIR and TITR. Age-related characteristics, including altered insulin sensitivity, delayed insulin absorption and reduced renal insulin clearance, may affect the effectiveness of more aggressive insulin administration in older adults (34). One of the most striking findings of our analysis is the distinct behavioral and algorithmic pattern observed between PwD achieving high versus lower TITR. The clear inverse relationship between the proportion of auto-correction insulin and standard bolus insulin underscores the vital role of user-driven meal management, even when utilizing advanced AID systems. In the lower (<50%) TITR subgroup, the significantly higher reliance on auto-corrections (averaging 20.6%) points toward a highly reactive pattern of glycaemic control. This elevated automated insulin delivery is consistent with a compensatory response of the algorithm to mitigate postprandial excursions caused by delayed, inadequate, or omitted meal boluses. While the hybrid closed-loop system acts as an excellent safety net to prevent extreme hyperglycemia, this reactive approach relies on insulin acting after glucose is already rising rapidly, which inherently limits the ability to maintain glucose levels within the strict 3.9–7.8 mmol/L target range. Conversely, the high (≥50%) TITR subgroup’s greater proportion of standard bolus insulin reflects proactive disease management. Accurate and timely—presumably pre-prandial—bolus administration covers carbohydrate intake before severe glycaemic excursions occur. Because the user is proactively managing mealtime spikes, the algorithm assumes its intended role of fine-tuning background insulin rather than repeatedly suppressing severe spikes. Consequently, the system is required to perform significantly fewer auto-corrections.

Several limitations of this study should be acknowledged. First, the number of participants using ROS was small, limiting statistical power to detect subtle differences in glycaemic control between groups. Second, the retrospective, cross-sectional design precludes causal inference and limits assessment of longitudinal effects following changes in algorithm settings. As this is a cross-sectional analysis, reverse causation cannot be excluded: as PwD who inherently maintain good glycaemic control likely experience less postprandial burden and therefore require fewer auto-corrections. Third, although algorithmic performance appears robust, effective use of AID systems also depends on user interaction with device interfaces. Older adults commonly experience cognitive and visual decline, known barriers to optimal diabetes technology use (8, 35). Notably, a selection bias may exist regarding which older adults are prescribed AID devices, meaning our results may not be fully generalizable to the broader, non-uniform population of older adult users. Furthermore, a significant proportion of these users rely heavily on younger spouses or other caregivers for their daily diabetes management, which may influence outcomes. Because this study relied on retrospective data extracted primarily via CareLink, nuanced geriatric and behavioral metrics—specifically cognitive/functional status, detailed living situations, hypoglycaemia awareness, and formal caregiver support—were not systematically recorded; therefore, a limitation of this study is that unmeasured psychosocial and functional factors may influence the observed behavioral patterns, such as manual bolus frequency. The inability of the retrospective CareLink database to reliably capture external clinical events, such as severe hypoglycaemia requiring assistance, serves as a limitation regarding the availability of clinical safety data. The marked female predominance in our cohort (70.1%) likely represents a selection bias regarding technology adoption, particularly given that cohorts with long-standing T1D (e.g., the Joslin Medallist study (36) with a mean age of 67.5 ± 7.5 years) showed a balanced ~50% sex distribution. This imbalance should be considered when generalizing these results.

Conversely, the inclusion of this understudied and heterogeneous population of older technology users is a primary strength of this study (37). Our research contributes to the evolving discourse on TIR and the necessary stringency of TBR targets for older adults. It also highlights TITR—a metric for which clinical guidelines do not yet provide specific parameters for this or any demographic (22, 24, 29, 32). There remains an ongoing clinical dilemma regarding the correlation between TIR and TBR; current evidence suggests that hypoglycemia targets may operate independently of TIR. Given the rising prevalence of T1D among older adults and the scarcity of evidence regarding aggressive algorithmic settings, further real-world evaluation is essential (22, 24). To optimize care for this aging population, forward-thinking strategies should include the development of care models that ensure continuity of technology use as users age, in addition to management strategies that account for the specific goals and capabilities of both the individual living with T1D and their care partners (17, 34).

Our findings underscore that while current hybrid closed-loop (HCL) systems provide a robust safety net against extreme glycaemic excursions, achieving superior metabolic control (with high TITR) remains heavily reliant on proactive user interaction. Our data suggest that the proportion of user-driven manual boluses—as opposed to reactive system auto-corrections—is a marker of greater engagement, thus suggesting that current fully closed-loop studies have so far shown limited success in achieving full control without meal announcement (38, 39). Current algorithms still rely on user interaction to maintain tight control during postprandial challenges (21, 25). Next-generation algorithms—potentially leveraging ultra-rapid-acting insulins, adjunctive therapies, or advanced predictive machine learning models—must be capable of autonomously detecting and aggressively managing mealtime excursions without any user input (38, 39). By removing the behavioral burden of meal announcements, fully automated systems may help bridge the precise performance gap observed in our cohort. Such advancements hold the potential to broaden access to tight glycemic control further, enabling all PwD to achieve exceptional metabolic outcomes (high TITR) independent of their age, engagement, health literacy, or technical adherence to the system (38, 40).

## 4 Conclusion

This real-world data analysis demonstrates that the use of the MiniMed™ 780G system in older adults with T1D is associated with low CGM-derived hypoglycemia exposure, even when using more aggressive algorithm settings. Since user engagement - specifically the administration of manual boluses – is associated with superior glycemic outcomes, the most critical future direction in AID development might be the transition to fully automated, closed-loop systems.

## Data Availability

The raw data supporting the conclusions of this article will be made available by the authors, without undue reservation.
